# Effects of Increased 1-Aminocyclopropane-1-Carboxylate (ACC) Deaminase Activity in *Bradyrhizobium* sp. SUTN9-2 on Mung Bean Symbiosis under Water Deficit Conditions

**DOI:** 10.1264/jsme2.ME20024

**Published:** 2020-06-16

**Authors:** Sukanlaya Sarapat, Pongpan Songwattana, Aphakorn Longtonglang, Kamolchanok Umnajkitikorn, Teerayoot Girdthai, Panlada Tittabutr, Nantakorn Boonkerd, Neung Teaumroong

**Affiliations:** 1 School of Biotechnology, Institute of Agricultural Technology, Suranaree University of Technology, Nakhon Ratchasima 30000, Thailand; 2 Suranaree University of Technology Farm (SUT Farm), Suranaree University of Technology, Nakhon Ratchasima 30000, Thailand; 3 School of Crop Production Technology, Institute of Agricultural Technology, Suranaree University of Technology, Nakhon Ratchasima 30000, Thailand

**Keywords:** 1-aminocyclopropane-1-carboxylate (ACC) deaminase activity, ethylene, infection/nodulation, *Vigna radiata*, water deficit conditions

## Abstract

Bacteria exhibiting 1-aminocyclopropane-1-carboxylic acid (ACC) deaminase activity, which inhibits the biosynthesis of ethylene in higher plants, promote plant growth through the degradation of ethylene precursors, such as ACC. ACC deaminase activity in *Bradyrhizobium* sp. SUTN9-2 was enhanced by genetic engineering and adaptive laboratory evolution (ALE)-based methods. The transferal of a plasmid containing the *acdR* and *acdS* genes into SUTN9-2 was genetic engineering improved, while the ALE method was performed based on the accumulation of an adaptive bacterial population that continuously grew under specified growth conditions for a long time. ACC deaminase enzyme activity was 8.9–fold higher in SUTN9-2:pMG103::*acdRS* and 1.4–fold higher in SUTN9-2 (ACCDadap) than in the wild-type strain. The effects of increased activity were examined in the host plant (*Vigna radiata* (L.) R.Wilczek SUT1). The improved strains enhanced nodulation in early stage of plant growth. SUTN9-2:pMG103::*acdRS* also maintained nitrogen fixation under water deficit conditions and increased the plant biomass after rehydration. Changes in nucleotides and amino acids in the AcdS protein of SUTN9-2 (ACCDadap) were then investigated. Some nucleotides predicted to be located in the ACC-binding site were mutated. These mutations may have increased ACC deaminase activity, which enhanced both symbiotic interactions and drought tolerance and promoted recovery after rehydration more than lower ACC deaminase activity. Adaptive evolution represents a promising strategy for further applications in the field.

Microbial 1-aminocyclopropane-1-carboxilic acid (ACC) deaminase inhibits ethylene biosynthesis through the degradation of ACC, a precursor of ethylene in higher plants, which promotes plant growth, particularly under environmental stress conditions (Saleem *et al.*, 2007; Saraf *et al.*, 2010; Glick, 2014; Gamalero and Glick, 2015). Plants often respond to stress conditions by increasing the production of ethylene, which is caused by the stress-induced accumulation of ACC and referred to as stress ethylene. Large amounts of ethylene induce tissue damage and reduce plant growth, which ultimately result in yield losses (Apelbaum and Yang, 1981; Kacperska and Kubacka-Zębalska, 1989; Stearns and Glick, 2003; Dubois *et al.*, 2018). Therefore, bacteria exhibiting ACC deaminase activity may modulate the production of ethylene in plants at appropriate levels for growth. To investigate the mechanisms by which soil bacteria containing ACC deaminase decrease ethylene production in plants, Glick *et al.* (1998) created a model and proposed that ACC, a product of *S*-adenosyl-methionine (SAM) via the catalysis of ACC synthase activity in plant tissues, is absorbed by soil bacteria that live around the plant roots or inside plant tissues and is then degraded by the ACC deaminase activity of bacteria to α-ketobutyrate and ammonia. Therefore, ACC levels may be controlled upstream of ethylene production in the ethylene biosynthesis pathway, leading to a decrease in the negative effects associated with the high production levels of ethylene. In addition to the reductions in plant growth described above, the ethylene hormone as a factor has been shown to interfere with nodule formation in rhizobia-legume interactions. Endogenous ethylene production induced by the application of exogenous ACC or ethylene was found to reduce nodule numbers in several legume plants, such as *Medicago sativa*, *Pisum sativum*, *Lotus japonicus*, *Macroptilium atropurpureum*, and *Trifolium repens* (Goodlass and Smith, 1979; Peters and Crist-Estes, 1989; Lee and LaRue, 1992a; Lee and LaRue, 1992b; Penmetsa and Cook, 1997; Nukui *et al.*, 2000; Oldroyd *et al.*, 2001). In contrast, leguminous plants were treated with ethylene synthesis inhibitors, such as L-α-(2-aminocthoxyvinyl) glycine (AVG) and silver thiosulfate (STS), as well as in an ethylene-insensitive mutant plant of *Medicago truncatula* (named *sickle*). Furthermore, plants treated with ethylene synthesis inhibitors and those with the *sickle* mutation had higher nodule numbers than untreated and wild-type plants. Tittabutr *et al.* (2008) demonstrated that that ACC deaminase activity was enhanced by the transferal of a plasmid containing multiple copies of native *acdS* (*Rhizobium* sp. TAL1145) and exogenous *acdS* (*Sinorhizobium* sp. BL3) into the TAL1145 strain, which increased ACC tolerance and nodule numbers as well as the biomass of *Leucaena leucocephala* more than the parental and *acdS* mutant strains. Moreover, a mutant of the *Sinorhizobium* sp. BL3 strain that was defective in *acdS* exhibited weaker nodulation competitiveness than the wild-type strain in mung bean plants, while a strain with increasing copy numbers of acdRS showed a higher nodule occupancy than wild-type strain when inoculated at 1:1 ratio. A previous study reported the beneficial effects of ACC deaminase on nodule senescence (Tittabutr *et al.*, 2015b)

Bacteria exhibiting ACC deaminase activity have been suggested to prevent reductions in plant growth under water deficit conditions. *Pseudomonas* strains enhanced drought stress tolerance in mung bean (*Vigna radiata* L.) (Kumari *et al.*, 2016) and eliminated or attenuated ethylene stress caused by water deficits in several pea cultivars (Arshad *et al.*, 2008; Zahir *et al.*, 2008), which promoted plant growth under drought conditions. In addition to *Pseudomonas* strains, other rhizobacteria, such as *Enterobacter*, *Bacillus*, *Pantoea*, and *Ochrobactrum*, have also been suggested to promote plant growth and enhance drought tolerance in several plants, including velvet bean (*Mucuna pruriens* [L.] DC.) (Saleem *et al.*, 2018), mung bean (Silambarasan *et al.*, 2019), black gram (*Vigna mungo* L.), and garden pea (*P. sativum* L.) (Saikia *et al.*, 2018). Co-inoculations of rhizobia with rhizobacteria or plant growth-promoting rhizobacteria (PGPR) exhibiting strong ACC deaminase activity may synergistically promote rhizobium-legume interactions and also reduce stress-induced water deficit. *V. radiata* plants inoculated with *Bradyrhizobium* sp. PRC008 in combination with rhizobacteria (*Enterobacter* and/or *Chryseobacterium* strains) exhibiting strong ACC deaminase activities showed a greater plant biomass and less ethylene biosynthesis than those with a single rhizobium inoculation under water deficit irrigation (Tittabutr *et al.*, 2013). A previous study also demonstrated the beneficial effects of co-inoculations in pea plants exposed to drying soil conditions; plants co-inoculated with *Rhizobium leguminosarum* bv. viciae and *Variovorax paradoxus* 5C-2 (exhibiting ACC deaminase activity) maintained or had higher nodule numbers and yield than plants not inoculated with this combination (Belimov *et al.*, 2009).

Belimov *et al.* (2019) recently reported that *R. leguminosarum* bv. viciae 1066S exhibiting ACC deaminase activity increased shoot biomass, nodulation, nitrogen fixation, water use efficiency (WUE), and nutrient uptake in pea plats exposed to water deficit conditions. Furthermore, *Rhizobium* strains exhibiting ACC deaminase enzyme activity enhanced soybean (*Glycine max* L.) germination under drought conditions, which was induced by 4% polyethylene glycol (PEG) (Igiehon *et al.*, 2019). Thus, the activity of ACC deaminase plays crucial roles in plant growth and root infection, particularly under unfavorable conditions. However, rhizobia exhibiting ACC deaminase activity have been less investigated in the aspect of ACC deaminase in correlating with ethylene production, nitrogen fixation, and the plant biomass after rehydration in mung bean plants under water deficit conditions.

The use of genetic engineering to increase ACC deaminase enzyme activity represents a promising strategy, but the improvement faces a limitation of releasing genetically modified organisms (GMO) Thailand, and it is still prohibited. In the present study, we designed two strategies to increase ACC deaminase activity by *Bradyrhizobium* sp. SUTN9-2. One strategy was genetic engineering, which involved the transferal of a plasmid containing the *acdR* and *acdS* genes into SUTN9-2. The *acdR* and *acdS* genes encode for the ACC deaminase regulator (AcdR) protein and ACC deaminase structural (AcdS) protein, respectively, which are responsible for synthesizing the enzyme ACC deaminase (Grichko and Glick, 2000; Li and Glick, 2001; Glick *et al.*, 2007). These two genes are present in opposite directions on a chromosome of SUTN9-2 (Piromyou *et al.*, 2015; 2017). The AcdR protein is a leucine-responsive regulatory (Lrp) protein, which is a specific regulator of *acdS* gene expression. The other strategy was the adaptive laboratory evolution (ALE) approach, which involves changing the metabolism of a microorganism without the use of a plasmid or addition of a foreign or heterologous gene into bacterial cells, but the bacteria cells were collected with growth-based selection under specific conditions (Dragosits and Mattanovich, 2013). Therefore, the objectives of the present study were to investigate the effects of increases in ACC deaminase activity by genetic engineering and ALE-based methods on symbiosis in mung bean plants, and also to assess its efficiency for the attenuation of ethylene stress induced by water deficit conditions. Amino acid changes in ACC deaminase derived from ALE were also investigated.

## Materials and Methods

### Increases in ACC deaminase activity in the SUTN9-2 strain

#### (i) ALE approach

**Fig. 1.**
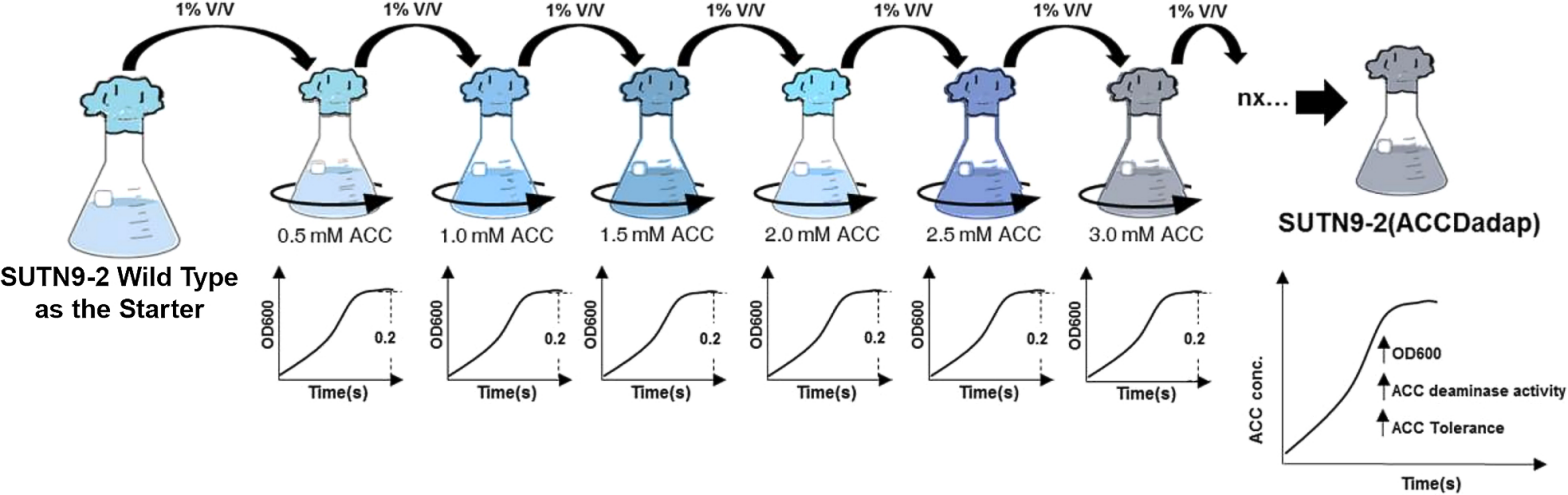
Increases in ACC deaminase activity by *Bradyrhizobium* sp. SUTN9-2. ACC deaminase in SUTN9-2 (WT) was improved by adaptive laboratory evolution (ALE). This approach was conducted based on sequential serial passages under a compulsive condition of ACC at 0.5, 1.0, 1.5, 2.0, 2.5 until 3.0‍ ‍mM. Optical density, ACC deaminase activity, and ACC tolerance were investigated to identify strains with increased ACC deaminase activity. The improved strain is named SUTN9-2 (ACCDadap).

To increase ACC deaminase activity using the ALE approach, a starter culture of *Bradyrhizobium* sp. strain SUTN9-2 (Piromyou *et al.*, 2015) (1% of the starter [v/v]) was cultured in minimal medium broth supplemented with 10% (v/v) of HEPES-MES (HM) broth and 0.5‍ ‍mM ACC (Sigma-Aldrich) to propagate the first starter culture of the SUTN9-2 adaptive strain. Bacteria were cultured at 30°C and 200 rpm until optical density (OD) reached 0.2 at 600 nm before being used as the next starter. Similarly, microbial cells (1% [v/v]) were cultured in minimal medium broth supplemented with 10% (v/v) HM broth containing 1.0‍ ‍mM ACC (final concentration) to create a new propagation, which was then cultured at 30°C and 200 rpm until OD reached 0.2 at 600 nm. Higher ACC concentrations of 1.5, 2.0, 2.5, and 3.0‍ ‍mM were similarly used after microbial cells reached 0.2 at 600 nm in parallel serial cultures (Fig. 1). Increases stopped at 3.0‍ ‍mM ACC (based on ACC deaminase activity) and the bacterial strain was named SUTN9-2 (ACCDadap).

#### (ii) Plasmid construction and transformation

The full fragment of *acdRS* (1,804 base pair) was amplified from the SUTN9-2 genome using the acdRXbaI-f (5′-TGCATCTCTAGAATCTCTCATGATGCGGATAGC-3′) and acdSBamHI-r (5′-CTGGCAAGGATCCGTCCTCGCAAAACGATTGCT-3′) primers. PCR conditions were as follows: 1 cycle at 95°C for 3‍ ‍min for the initial denaturation step; 35 cycles at 95°C for 1‍ ‍min, 55°C for 1‍ ‍min, and 72°C for 1‍ ‍min, and 1 cycle at 72°C for 5‍ ‍min (T100^TM^ Thermal Cycler; Bio-Rad Laboratories). The fragment of *acdRS* was digested by *Xba*I and *Bam*HI and ligated into pMG103-nptII-cefo^r^ (Wongdee *et al.*, 2016) at complementary sites to obtain the recombinant pMG103::*acdRS*-S9-2::nptII-cefo^r^ plasmid (Fig. S1). This recombinant plasmid was transferred into *Escherichia coli* (DH5α) and spread on LB containing 20‍ ‍μg mL^–1^ cefotaxime for selection. pMG103::*acdRS*-S9-2::nptII-cefo^r^ was then extracted using the alkaline lysis method (Bimboim and Doly, 1979) and directly transformed into competent cells of SUTN9-2. Competent cells were prepared by culturing SUTN9-2 in YEM broth at 30°C with shaking at 200 rpm until bacterial cells reached 0.4 at OD600, and cells were collected by centrifugation at 5,000 rpm and 4°C for 15‍ ‍min. After discarding the medium, sterile deionized water was used for washing (3 times) followed by 10% glycerol (twice). Competent cells were immediately stored at –80°C or used freshly after cells had been resuspended in 10% glycerol. The transformation of the plasmid was conducted by electroporation (15 kv cm^–1^, 100‍ ‍Ω, and 25 μF; BTX^TM^ Gemini X2 Electroporation System). Colonies of SUTN9-2 containing pMG103::*acdRS*-S9-2::nptII-cefo^r^ were cultured on YM supplemented with 20‍ ‍μg mL^–1^ cefotaxime. SUTN9-2 harboring the pMG103::*acdRS*-S9-2::nptII-cefo^r^ plasmid was named SUTN9-2:pMG103::*acdRS*.

### Bacterial culture conditions and bacterial cell preparations

*Bradyrhizobium* sp. SUTN9-2 (WT), SUTN9-2 (ACCDadap), and SUTN9-2:pMG103::*acdRS* were cultured in 100‍ ‍mL HEPES-MES (HM) salt medium broth (Cole and Elkan, 1973; Kanbe *et al.*, 2007) at 30°C and 200 rpm with shaking for 7‍ ‍d prior to use. The medium used for the growth of the SUTN9-2:pMG103::*acdRS* strain was HM supplemented with 20‍ ‍μg mL^–1^ cefotaxime. To induce ACC deaminase expression, bacterial cells were transferred into minimal medium (Soedarjo *et al.*, 1994; Tittabutr *et al.*, 2008) supplemented with 10% (v/v) HM and 3.0‍ ‍mM ACC and then incubated at 30°C and 200 rpm for 24 h (Siddikee *et al.*, 2011).

### ACC deaminase activity measurements

Bacterial cells were cultured in 15‍ ‍mL HM broth at 30°C and 200 rpm with shaking for 7‍ ‍d until they reached the early stationary phase. Bacterial cells were collected by centrifugation at 5,000 rpm and 25°C for 15‍ ‍min and washed twice with minimal medium. Bacterial cells were resuspended in 15‍ ‍mL of minimal medium supplemented with 10% (v/v) HM containing 1‍ ‍mM ACC, and then incubated at 30°C and 200 rpm for 40 h. Enzyme activity was assessed as described by Tittabutr *et al.* (2008). Protein levels were measured using Lowry’s method (Lowry *et al.*, 1951).

### Investigation of ACC tolerance

Bacterial cells (0.1% [v/v]) including SUTN9-2 (WT), SUTN9-2 (ACCDadap), and SUTN9-2:pMG103::*acdRS* were cultured in 3‍ ‍mL yeast extract-mannitol (YEM) broth (Somasegaran and Hoben, 1985) containing ACC at a final concentration of 3.0‍ ‍mM after normalization by OD600 nm reaching 1.0. Bacterial cultures were cultivated at 30°C without any shaking for 15 d. Viable cells were assessed by a viable plate count on YEM medium.

### Plant material and inoculation of bacterial strains

Seeds of *Vigna radiata* (L.) R. Wilczek SUT1 were surface sterilized with 95% (v/v) ethanol for 10‍ ‍s followed by 3% (v/v) sodium hypochlorite for 5‍ ‍min and washed 5–10 times with sterilized distilled water. Seeds were soaked overnight in sterilized distilled water. Seeds were germinated on sterilized wet tissue paper at room temperature for 2 d. Seedlings (2 plants jar^–1^) were grown in modified Leonard jars, which contained sterilized sand, and were watered with a nitrogen-free nutrient solution (Somasegaran and Hoben, 1985). Seedlings were grown at 28±2°C and 60% relative humidity with a 12-h light/dark cycle under a white fluorescent light (approximately 300 μE m^–2^ S^–1^). Before the inoculation, bacterial cells were washed twice with sterilized 0.03 M MgSO_4_, and 1‍ ‍mL of each bacterial culture after adjustments to the same optical density (OD_600_=1.0) was then inoculated into 2-day-old seedlings of mung bean. Twelve days after inoculation (DAI), the nodules and plant biomasses of mung bean were dried at 70°C for 48 h and then recorded.

### Water deficit and rehydration conditions

Mung bean seedlings were grown under normal conditions for two weeks after sowing, and water deficit conditions were achieved by discarding nitrogen-free nutrient solution from Leonard jars under the same normal conditions of 28±2°C and 60% relative humidity with a 12-h light/dark cycle under a white fluorescent light (approximately 300 μE m^–2^ S^–1^). Mung bean plants exhibiting wilt symptoms 7‍ ‍d after water had been discarded were harvested. Ethylene emissions from each plant were measured by gas chromatography (GC) (Model 310; SRI Instruments) equipped with a flame ionization detector (6'×1/8" SS column; Valco Instruments) according to the method described by Tittabutr *et al.* (2013). To assess the effects of bacteria exhibiting ACC deaminase activity on plant growth after rehydration conditions, mung bean seedlings were exposed to water deficit conditions at initial planting in modified Leonard jars. The progressive drying of sand was controlled at 10% of the permanent wilting point (PWP) by adding 1‍ ‍mL of nitrogen-free nutrient solution into sand to control its humidity. at 12 DAI, mung bean plants were rehydrated with nitrogen-free nutrient solution for twenty d under the same conditions as those described above and recovered plants were harvested at 32 DAI.

### Nitrogenase activity measurement

The detached root nodules of mung bean plants were incubated in 15-mL rubber-capped plastic tubes from which 5% (v/v) of air was withdrawn and replaced with pure acetylene. Samples were incubated at 25°C for 1 h for the conversion of acetylene to ethylene. One milliliter of airspace was injected into the GC machinery to measure ethylene gas. Nitrogenase activity was shown as nmol ethylene h^–1^ mg^–1^ nodule dry weight (Somasegaran and Hoben, 1985).

### Investigation of nucleotides, annotated amino acid sequences, and three-dimensional structures of ACC deaminase enzymes

The nucleic acid sequences of *acdR* and *acdS* in the SUTN9-2 (ACCDadap) strain were amplified using PCR as described above. The PCR product was ligated into the pTG19-T PCR cloning vector (Vivantis Technologies Sdn Bhd) for sequencing after transformation in the *E. coli* strain DH5α and plasmid extraction. The DNA sequences of *acdR* and *acdS* containing the vectors were elucidated by Macrogen (Korea). These sequences were analyzed by alignment using multiple sequence alignment with hierarchical clustering (Corpet, 1988), a published piece of software (http://multalin.toulouse.inra.fr/multalin/), and were compared with the nucleic acid and amino acid sequences of the SUTN9-2 (WT) strain. The protein structure of ACC deaminase in the SUTN9-2 (ACCDadap) strain was predicted and created using Phyre^2^ software (Kelley *et al.*, 2015; Ittisoponpisan *et al.*, 2019) and the PyMOL Molecular Graphics System. The 3DLigandSite-Ligand binding site prediction server (Wass *et al.*, 2010) was used to predict the binding sites of ACC deaminase in the SUTN9-2 (ACCDadap) strain compared with ACC deaminase in SUTN9-2 (WT), *Pseudomonas* sp. ACP, and *P. putida* UW4 strains.

### Statistical analysis

All parameters were analyzed by SPSS program version 16.0 (SPSS) for Windows software to identify differences between means at *P*≤0.05. A one-way analysis was conducted for comparisons between treatments, and Duncan’s multiple range tests were used to assess the significance of differences among means. Data are shown as the mean±S.E. calculated from three and five replicates. Moreover, data were transformed when normality was not achieved.

## Results

### ACC deaminase activities of SUTN9-2 (ACCDadap) and SUTN9-2:pMG103::acdRS strains

ACC deaminase activity in plants inoculated with SUTN9-2 (WT) was 3.88 μmol of α-ketobutyrate h^–1^ mg^–1^ protein, and was higher (1.4-fold that of wild type, 5.58‍ ‍μmol of α-ketobutyrate h^–1^ mg^–1^ protein) in plants inoculated with SUTN9-2 (ACCDadap) and significantly higher (8.9-fold that of wild type, 34.72 μmol of α-ketobutyrate h^–1^ mg^–1^ protein) in those inoculated with SUTN9-2:pMG103::*acdRS* (Table 1). To confirm that enzyme activity correlated with ACC tolerance, the survival of the improved strains at the high ACC concentration of 3.0‍ ‍mM was examined. The results obtained showed that colony-forming units (CFU) were significantly higher for the improved strains than for SUTN9-2 (WT); by up to 1.21-fold for SUTN9-2:pMG103::*acdRS* and 1.08-fold for SUTN9-2 (ACCDadap) after they had been cultured for 15‍ ‍d in YEM supplemented with 3.0‍ ‍mM ACC (Table S1).

### Effects of *Bradyrhizobium* sp. SUTN9-2 exhibiting increased ACC deaminase activity on mung bean symbiosis

**Fig. 2.**
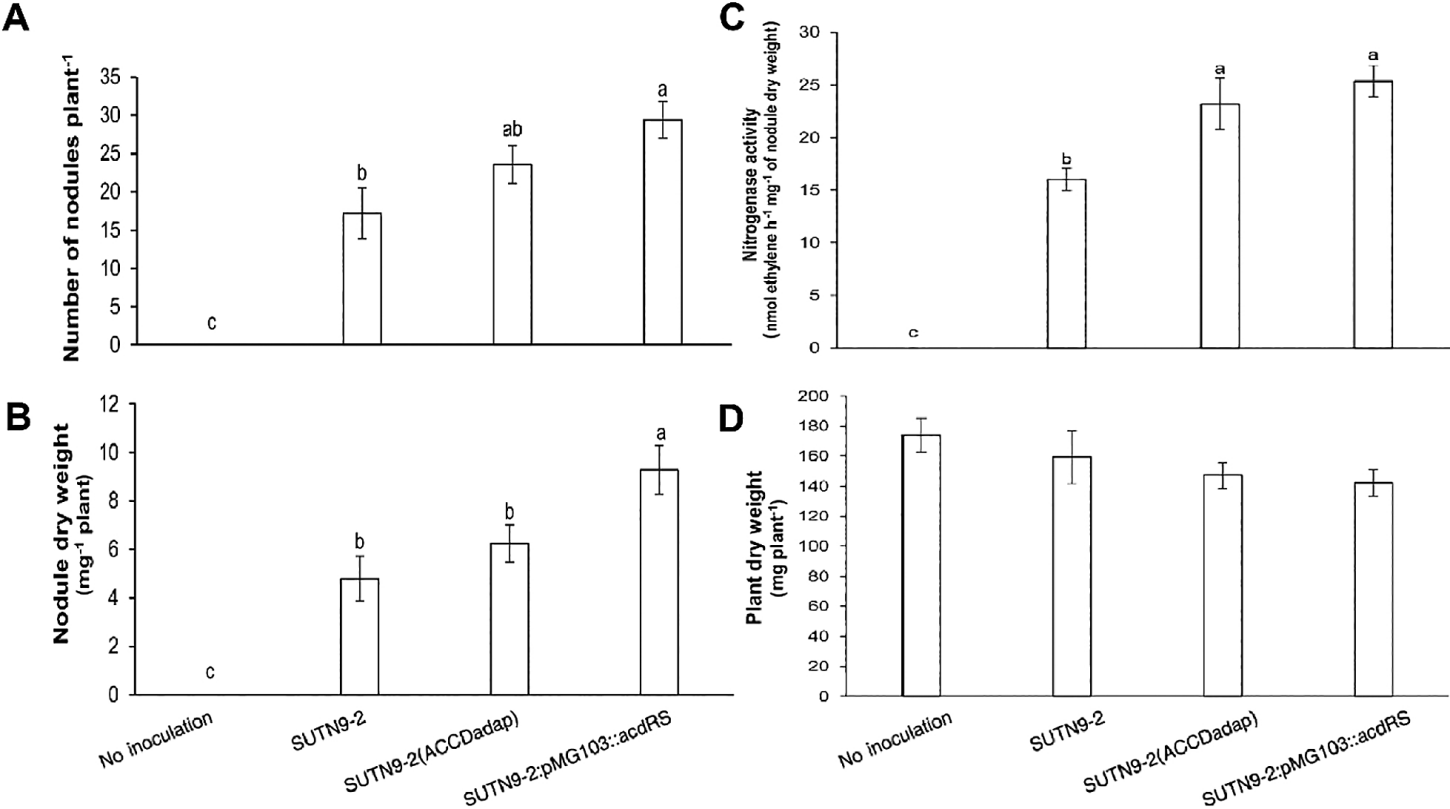
The average (A) number of nodules, (B) nodule dry weight, (C) nitrogenase activity, and (D) plant dry weight of mung bean plants at 12 DAI inoculated with *Bradyrhizobium* sp. SUTN9-2 and its modified strains, SUTN9-2 (ACCDadap) and SUTN9-2:pMG103::*acdRS* and of plants that were not inoculated. Data are presented as the mean of seven replicates and the vertical bars indicate standard errors. Significant differences were shown with different letters at *P*≤0.05 according to Duncan’s multiple range tests.

Since ethylene inhibits nodule formation, particularly in the early process of infection, mung bean plants were inoculated with the improved and wild-type strains. The results obtained revealed that the nodule numbers of mung bean plants inoculated with SUTN9-2 (ACCDadap) and SUTN9-2:pMG103::*acdRS* strains were up to 1.4-fold and 1.7-fold higher, respectively, than those inoculated with SUTN9-2 (WT) (Fig. 2A). The nodule dry weights of plants‍ ‍inoculated with SUTN9-2 (ACCDadap) and SUTN9-2:pMG103::*acdRS* were higher (up to 1.3-fold) and‍ ‍significantly higher (up to 1.9-fold), respectively, than‍ ‍those inoculated with SUTN9-2 (WT) (Fig. 2B). Furthermore, nitrogenase activity was up to 1.5-fold higher with SUTN9-2 (ACCDadap) and 1.6-fold higher with SUTN9-2:pMG103::*acdRS* that that with SUTN9-2 (WT) (Fig. 2C). However, no significant differences were observed in the plant dry weights of mung bean plants inoculated with the improved strains and SUTN9-2 (WT) (Fig. 2D).

### Efficiency of SUTN9-2 containing modified ACC deaminase strains against water deficit and rehydration conditions

**Fig. 3.**
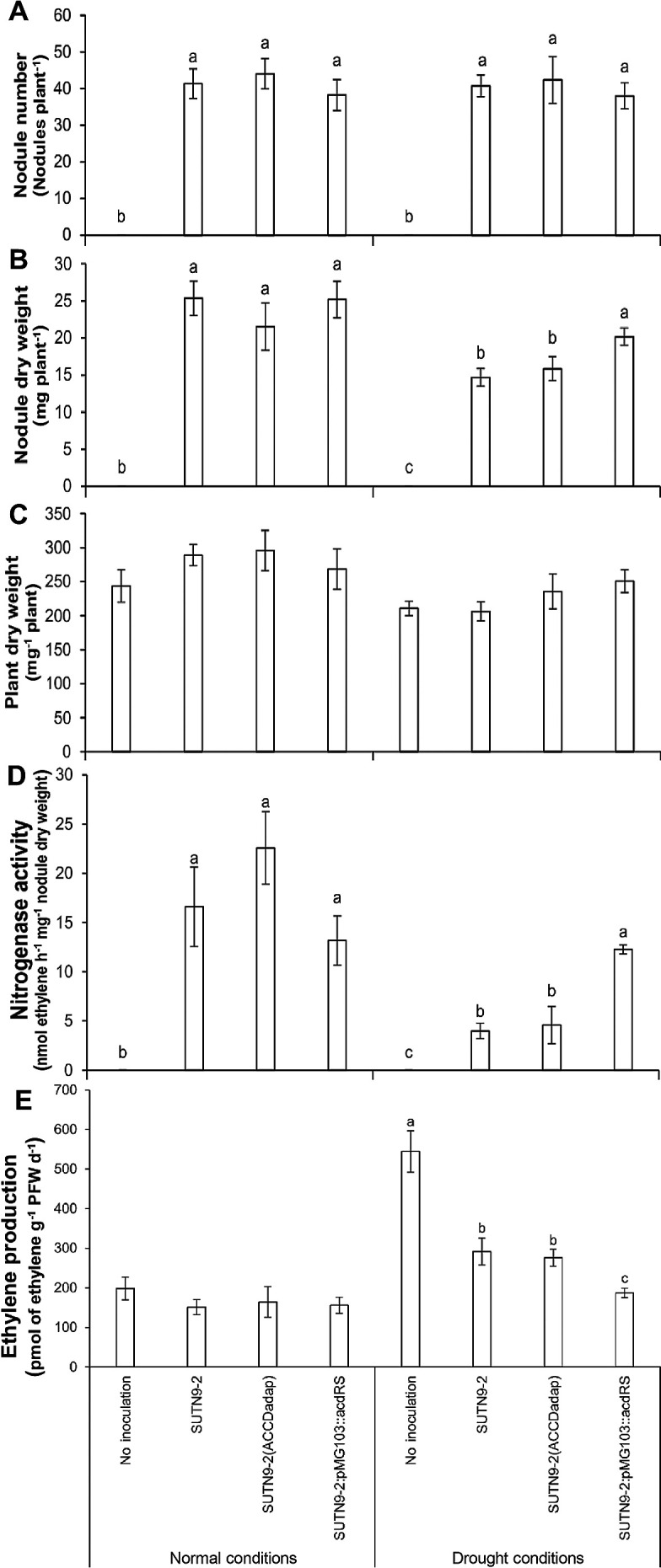
The average (A) number of nodules, (B) nodule dry weight, (C) plant dry weight, (D) nitrogenase activity and (E) ethylene production of mung bean plants at 19 DAI inoculated with *Bradyrhizobium* sp. SUTN9-2 and its modified strains, SUTN9-2 (ACCDadap) and SUTN9-2:pMG103::*acdRS* and of plants that were not inoculated under normal and drought conditions. Data are presented as the mean of seven replicates, and the vertical bars indicate standard errors. Significant differences were shown with different letters at *P*≤0.05 according to Duncan’s multiple range tests. Comparisons were performed separately for normal and drought conditions.

**Fig. 4.**
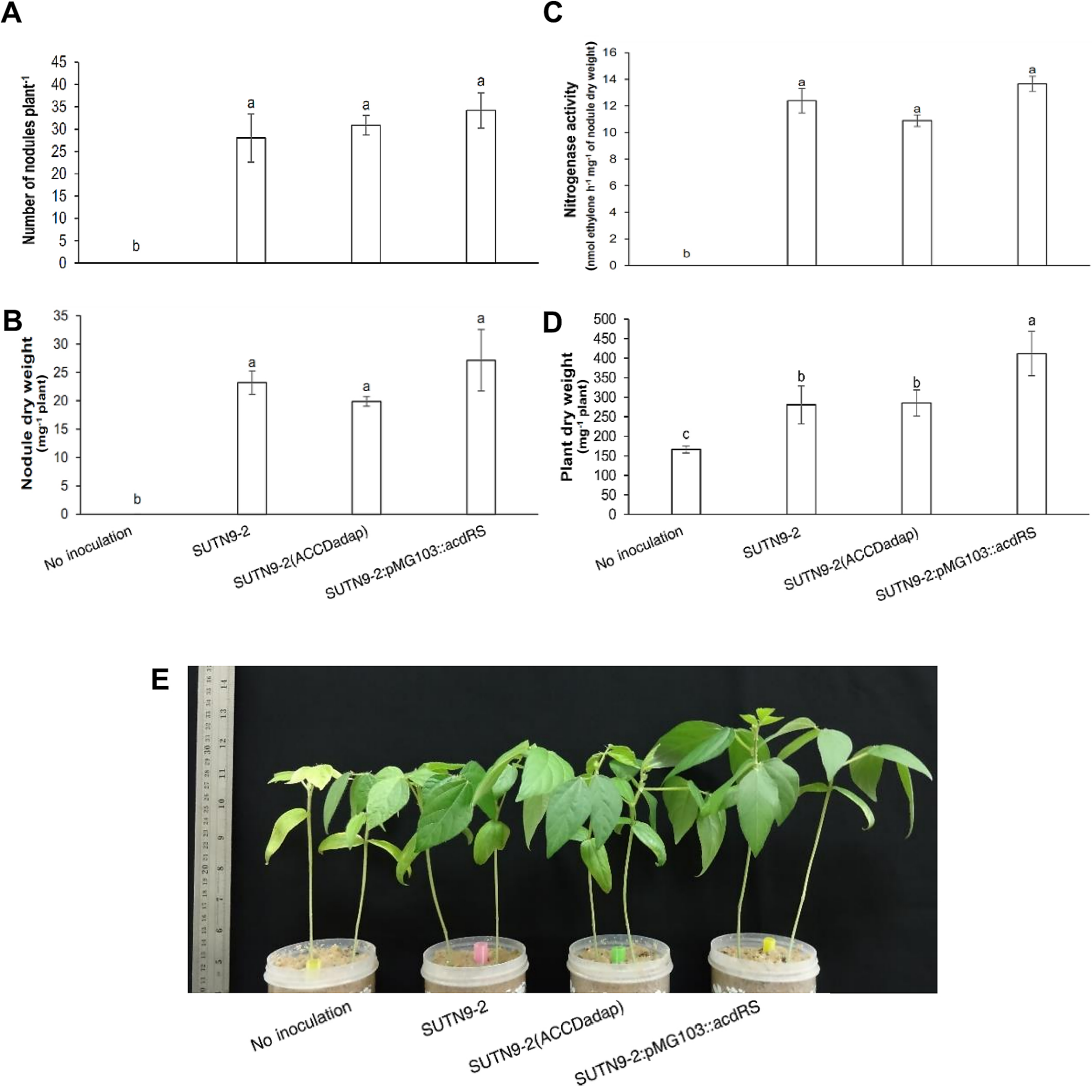
Effects of bacteria exhibiting ACC deaminase activity and the influence of increased ACC deaminase activity on mung bean plants after rehydration for 20 d. The average (A) number of nodules, (B) nodule dry weight, (C) nitrogenase activity, (D) plant dry weight, and (E) phenotypes of mung bean plants at 32 DAI inoculated with *Bradyrhizobium* sp. SUTN9-2 and its modified strains, SUTN9-2 (ACCDadap) and SUTN9-2:pMG103::*acdRS *and of plants that were not inoculated under normal and deficit conditions. Data are presented as the mean of seven replicates and the vertical bars indicate standard errors. Significant differences were shown with different letters at *P*≤0.05 according to Duncan’s multiple range tests.

To assess the efficiency of increased ACC deaminase activity against water deficit stress on mung bean growth and its nitrogenase activity, watering was stopped at 12 DAI and plant morphology was observed. The results obtained indicated that the nodule number of plants inoculated with each bacterial strain did not significantly differ under normal or water deficit conditions (Fig. 3A). However, nodule dry weight, plant dry weight, and nitrogen fixation were lower under water deficit conditions than under normal conditions (Fig. 3B, C, and D). Under water deficit conditions, no significant differences were noted in plant dry weight; however, plant growth (nodule and plant dry weights) was slightly better by plants inoculated with the improved strains (Fig. 3B and C) than by those inoculated with the wild-type strain or not inoculated. SUTN9-2:pMG103::*acdRS* retained higher nitrogenase activity than SUTN9-2 (ACCDadap) under water deficit conditions, while SUTN9-2 (ACCDadap) promoted nitrogen fixation more than SUTN9-2:pMG103::*acdRS* under normal conditions (Fig. 3D). High ethylene emission levels were detected under water deficit conditions, particularly from plants that were not inoculated, while all plants inoculated with bacteria produced significantly lower levels of ethylene than non-inoculated plants. The lowest ethylene emission level was detected in plants inoculated with SUTN9-2:pMG103::*acdRS*. No significant differences were observed in ethylene emission levels among plants grown under normal conditions, and ethylene emission levels were lower than those by plants grown under water deficit conditions (Fig. 3E). Increased ACC deaminase activity in rhizobium SUTN9-2:pMG103::*acdRS* may have increased plant dry weight significantly more than the other treatments after rehydration (Fig. 4D), which was consistent with the plant phenotypes (Fig. 4E). However, no significant differences were found in nodule numbers, nodule dry weight, or nitrogen fixation among the three inoculated plants (Fig. 4A, B, and C).

### Influence of ALE on *acdS* and its protein structure

**Fig. 5.**
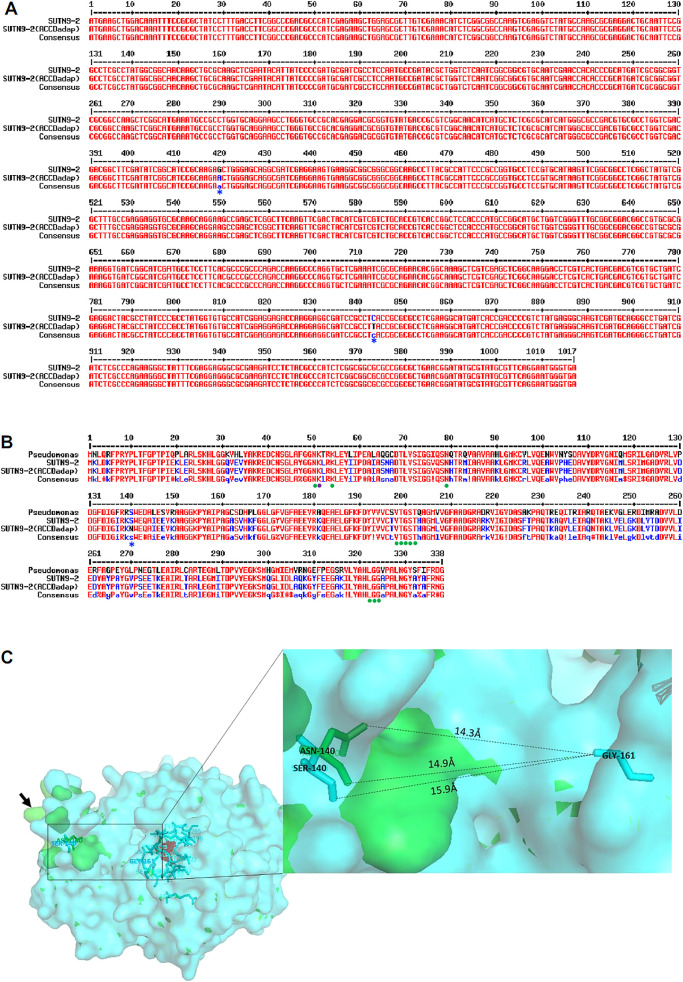
Sequence alignments of nucleotides and amino acids of the *acdS* gene that encodes the ACC deaminase enzyme. (A) Alignment comparison of the nucleotide sequences of the *acdS* gene containing SUTN9-2 (ACCDadap) with SUTN9-2 (WT). The blue asterisk (*) is a nucleotide sequence that changed from SUTN9-2 (WT) located at positions 419 and 843. (B) A comparison alignment of amino acid sequences of ACC deaminase containing *Bradyrhizobium* sp. SUTN9-2 (ACCDadap) with SUTN9-2 (WT). The Lys^51^ residue is marked with a purple circle as a binding site of PLP. The green circles were the predicted binding sites of ACC deaminase-containing SUTN9-2 (ACCDadap) and its wild-type strain *P. putida* UW4 and *Pseudomonas* sp. ACP. The blue asterisk (*) is the position of an amino acid that changed from a serine (Ser/S) in SUTN9-2 (WT) and *Pseudomonas* sp. strains to an asparagine (Asn/N) in SUTN9-2 (ACCDadap) at position 140. (C) The superposition of the three-dimensional structure of ACC deaminase-containing SUTN9-2 (WT) (aqua color) and SUTN9-2 (ACCDadap) (green color). The arrow points to the folding of the ACC deaminase structure, which changed from a native structure (aqua color) to a modified structure (green color) as a result of the asparagine (Asn/N) at position 140 impacting on the folding of the GLY^161^ amino acid residue, leading to its prediction as a binding site (green color).

The sequence alignment of the *acdS* gene in SUTN9-2 (ACCDadap) showed some changes in its nucleic acid sequence encoding the ACC deaminase enzyme (Fig. 5A). The guanine base (G), located at position 419 of the *acdS* gene in SUTN9-2 (WT), was changed to an adenine base (A) in SUTN9-2 (ACCDadap). The cytosine base (C) located at position 843 in SUTN9-2 (WT) was changed to a thymine base (T) in SUTN9-2 (ACCDadap). The alteration in the nucleic acid sequence at position 419 in SUTN9-2 (ACCDadap) affected the encoding of amino acid residues. The DNA base sequence of the *acdR* gene in SUTN9-2 (ACCDadap) did not appear to differ from that of SUTN9-2 (WT) (data not shown). The results of protein residue alignments demonstrated that the amino acid residues encoding ACC deaminase in SUTN9-2 (ACCDadap) showed 99% identity with those in SUTN9-2 (WT). This result may be explained by changes in the nucleic acid sequence from G to A at position 419 of the *acdS* gene. This modification caused a change in the serine (Ser/S) protein residue in SUTN9-2 (WT) to an asparagine (Asn/N) residue in SUTN9-2 (ACCDadap) at position 140 of the amino acid residue encoding ACC deaminase (Fig. 5A and B). However, the change at position 843 of the nucleic acid sequence did not affect encoding of the amino acid residue of ACC deaminase enzyme in SUTN9-2 (ACCDadap). Furthermore, changes in the amino acid residue encoding ACC deaminase enzyme in SUTN9-2 (ACCDadap) altered the configuration of the enzyme. These results showed that alpha helix and beta strand structures accounted for 43 and 15%, respectively, of the protein structure of ACC deaminase (AcdS protein) in SUTN9-2 (ACCDadap), and 42 and 16%, respectively, of that in SUTN9-2 (WT). Moreover, the AcdS protein sequence in SUTN9-2 (WT) shared sequence identities of 71 and 70% with *Pseudomonas* sp. ACP and *P. putida* UW4, respectively (Fig. S2).

Changes in amino acid residues as a result of alterations in nucleotide sequences led to enrichment modifications in the binding site of the protein structure of ACC deaminase enzyme in SUTN9-2 (ACCDadap). Collectively, the results obtained on the predicted binding site showed that SUTN9-2 (ACCDadap) possessed a new predicted binding site as a glycine positioned on residue 161 (Gly^161^), which was not observed in SUTN9-2 (WT) (Table. S2). Therefore, changes in the amino acid residue, asparagine, located at position 140 (Asn^140^), may have had an impact on the formation or folding of the protein structure (Fig. 5C).

## Discussion

Bacteria exhibiting ACC deaminase activity have been divided into two general types: symbiotic relationships and free-living microorganisms. Symbiotic bacteria exhibit lower ACC deaminase activity than free-living microorganisms (Glick, 2005, 2014). SUTN9-2 exhibited ACC deaminase activity of approximately 3.48 μmol of α-ketobutyrate h^–1^ mg^–1^ protein, which is lower than that of *P. putida* UW4 (20.48 μmol of α-ketobutyrate h^–1^ mg^–1^ protein) acting as a free-living bacterial strain (Ma *et al.*, 2003). The present study described approaches for increasing ACC deaminase activity using the ALE strategy (SUTN9-2 [ACCDadap] strain) compared with a h^–1^ mg^–1^ protein. Under continuous evolution, the SUTN9-2 (ACCDadap) strain was accumulated and selected at a high ACC concentration (3‍ ‍mM) as a specified growth condition, leading to better bacterial growth and ACC tolerance. This phenomenon based on the life cycle of bacteria indicate that the prolonged periods in stationary phase or starvation period are a duration of mutant selection with increasing the fitness express growth advantage in stationary phase (GASP), and then they can grow and scavenge the parent cells, ultimately as a main population (Bačun-Družina *et al.*, 2011). The increased ACC deaminase activities exhibited by the improved strains, SUTN9-2 (ACCDadap) and SUTN9-2:pMG103::*acdRS*, were confirmed not only by measurements of α-ketobutyrate (Table 1), but also by ACC tolerance and/or the utilization of ACC. Since bacteria producing the ACC deaminase enzyme may use ACC as a source of nitrogen (Glick *et al.*, 1998; Vanderstraeten and Van Der Straeten, 2017), these findings confirm increased enzyme activities. ACC deaminase improved bacterial strains grew better than SUTN9-2 (WT), as shown by the increase in their cells at a high concentration of ACC during their growth in YEM medium which were less affected by ACC. These results indicated that our methods increased ACC deaminase activity and ACC tolerance over those in the wild-type strain based on the number of bacterial cells (Table S1 and Table 1). A high concentration of ACC is toxic to bacterial cells. However, we hypothesized that increased ACC deaminase activity increases the efficiency of cells to detoxify high ACC concentrations. The preliminary results of the present study showed that the growth of SUTN9-2 (WT) was limited by a high ACC concentration (3‍ ‍mM ACC), while strains with increased ACC deaminase activity tolerated high ACC concentrations better than SUTN9-2 (WT) based on viable cell numbers (Table S1).

ACC deaminase activity and expression levels play an important role in the nodulation process and symbiosis; the symbiont tolerates ethylene levels that affect infection, leading to increases in the number of nodules (Fig. 2A), nodule dry weight (Fig. 2B), and nitrogenase fixation (Fig. 2C). Direct evidence to confirm the effects of ACC deaminase activity on the nodulation process was provided by the results obtained from the deletion mutant of the gene encoding the enzyme ACC deaminase (*acdS*) as well as from the introduction of the exogenous *acdS* gene. Nodulation by the defective mutant strain in ACC deaminase was lower, while its nodulation ability in rhizobia containing an exogenous ACC deaminase gene was higher than that by the wild-type strain, which exhibited low or no ACC deaminase activity (Ma *et al.*, 2003; 2004). However, regarding the promotion of plant growth, no significant differences were observed in the plant biomass between mung bean plants inoculated or not with bacteria (Fig. 2D). Since the plants examined in this experiment were 12 DAI, nitrogen for plant growth may have been covered by nutrients in seeds; therefore, the bacterial inoculation did not affect the plant biomass.

Under normal conditions, increases in ACC deaminase activity by bacteria did not alter plant growth or symbiosis (Fig. 3). These results were consistent with previous findings reported by Ma *et al.* (2003), showing that the inoculation of plants with *R. leguminosarum* bv. viciae (high expression mutation of ACC deaminase activity) did not significantly increase nodule numbers, shoot dry weights, or nitrogenase activity over those in the wild-type strain under normal conditions. Increases in ACC deaminase activity reduced stress tolerance in the host plant, as demonstrated by the SUTN9-2:pMG103::*acdRS* strain efficiently increasing nodule dry weight and nitrogenase activity under stress conditions (Fig. 3B and D). However, nodule numbers (Fig. 3A) and plant dry weight (Fig. 3C) at 19 DAI were not affected by the increase in ACC deaminase activity under water deficit conditions.

The relationships between nodule senescence were derived from other factors including large amounts of reactive oxygen species (ROS) and decreases in antioxidants, such as ascorbate and glutathione, in nodules, which affected the accumulation of ROS (Puppo *et al.*, 2005). Plant hormones, including abscisic acid and ethylene, may regulate nodule senescence through carbon metabolism. Changes in the ratio of sugar/nitrogen in nodules is a signal for the activation of abscisic acid, which often occurs in plants. The rate of this change was supported by lower carbon and higher nitrogen metabolite availabilities in nodules (Tittabutr *et al.*, 2015a). Moreover, this change may induce nodule senescence, which resulted in reduced nitrogen fixation in mung bean plants inoculated with SUTN9-2:pMG103::*acdRS* at 19 DAI under normal conditions. Nodule senescence caused by environmental stress generally occurs faster than developmental senescence because ethylene, a senescence hormone, was found to be strongly induced under environmental stress conditions (Puppo *et al.*, 2005; Tittabutr *et al.*, 2015a). Previous studies found that water deficit conditions reduced the supply of carbon (malate). Thus, the carbon supply could not migrate into bacteroids because of photosynthesis reduction as a result of stomatal closure, leading to reductions in CO_2_ availability and transpiration (González *et al.*, 1998; Larrainzar *et al.*, 2009). These findings suggested that an insufficient supply of carbon metabolism directly affected nitrogen fixation and may also have induced ethylene biosynthesis (Gazzarrini and McCourt, 2001). Berrabah *et al.* (2018) demonstrated that exogenous ethylene (25 to 250,000 ppm) reduced nitrogen fixation by *M. truncatula* and *L. japonicas* after an incubation for 3‍ ‍d, but did not affect that by the *sickle* plant. These findings were attributed to ethylene stimulating plant defenses, resulting in necrotic areas in the central parts of the root nodules. In the present study, the nitrogenase activity of plants inoculated with bacteria exhibited lower nitrogenase activity under water deficit conditions, except for mung bean plants inoculated with SUTN9-2:pMG103::*acdRS*. Since SUTN9-2:pMG103::*acdRS* exhibited high ACC deaminase activity, leading to the moderation of nodule senescence through decreases in the substrate for ethylene synthesis, plants inoculated with SUTN9-2:pMG103::*acdRS* exhibited high nitrogenase activity under water deficit conditions (Fig. 3D and E).

SUTN9-2:pMG103::*acdRS* increased plant dry weights after rehydration (Fig. 4D). Staudinger *et al.* (2016) proposed that *M. truncatula* inoculated with *S. medicae* WSM419 exhibiting ACC deaminase activity had lower levels of the proteins involved in ethylene synthesis, such as ACC oxidase, which converts ACC to ethylene in the ethylene biosynthesis pathway, than plants not inoculated under water deficit conditions. This reduction in ethylene production delayed leaf senescence, resulting in less leaf abscission. Their findings also suggested that bacteria-inoculated plants were provided with a carbon source in the form of sugar rather than starch. Therefore, the rehydration of plants inoculated with bacteria was faster than that of plants not inoculated because the utilization of the sugar form as a carbon source was easier than that of starch. Moreover, the accumulation of the sugar form persisted after rehydration, which did not occur in plants that were not inoculated. Therefore, the nodulation of rhizobacteria may facilitate recovery by controlling plant metabolism, such as plant hormones, the proportion of anions/cations, C and N stabilization, and the ability to change proteins. Irigoyen *et al.* (1992) demonstrated that the amount of ethylene emission was increased in correlation with the severity level of water deficit, which often accompanied by increase lipid peroxidation and hydrogen peroxide (H_2_O_2_) production in alfalfa. Therefore, bacteria exhibiting ACC deaminase activity may decrease H_2_O_2_ levels to induce nodule senescence. These findings support the overproduction of ACC deaminase activity in SUTN9-2:pMG103::*acdRS* retaining nitrogenase activity under drought stress conditions. Hence, we suggested that increases in ACC deaminase activity using genetic engineering were more efficient than those by adaptive evolution for enhancing nodulation at the seedling stage and alleviation drought stress conditions. However, the adaptive evolution strain may promote nitrogen fixation under normal conditions.

The ALE approach increased ACC deaminase activity by changing the DNA sequence of the *acdS* gene, but not the nucleotide sequence of the *acdR* gene. Previous studies reported that AcdR is a transcriptional regulatory protein of *acdS* that synthesizes the ACC deaminase enzyme when it is induced by ACC (Grichko and Glick, 2000; Leonard *et al.*, 2001; Li and Glick, 2001; Glick *et al.*, 2007). Therefore, under the ALE approach by which the mutant was selected based on increasing the fitness express growth with ACC, the AcdR protein needed to be stable. Since the stability of the *acdR* gene resulted in ACC deaminase expression, the nucleotide sequence of the *acdR* gene present in SUTN9-2 (ACCDadap) may not be modified. Thus, a change in the nucleotide sequence of *acdS* may have had a greater impact on increases in ACC deaminase activity than *acdR* in the present study. Moreover, the protein three-dimensional structure was used as evidence to confirm the hypothesis that can be explained by new distance (14.3 Å and 14.9 Å) of amino acid residues between Asn^140^ (green color) and Gly^161^ (aqua color) containing SUTN9-2 (ACCDadap) while SUTN9-2 (WT) showed the further distance (15.9 Å) between Ser^140^ (aqua color) and Gly^161^ (aqua color) (Fig. 5C). In addition, the molecular weight of an asparagine (Asn) is higher (residue mass) than that of a serine (Ser), which was located at position 140. Therefore, we suggested that amino acid residues had an impact on the formation or folding of the protein structure (Gly^161^). Moreover, the three-dimensional structure showed the predicted binding site as Gly^161^ located at a small domain fold of ACC deaminase, which lies on the front side of the pyridine in the loop of the binding site, which is beneficial for ACC binding and increasing ACC deaminase activity (Yao *et al.*, 2000; Karthikeyan *et al.*, 2004). The results of three-dimensional alignments showed that protein structures differed from the protein residues of bacteria (Fig. S3A). Differences in protein structures were shown by colored lines, which did not overlap in comparison with ACC deaminase structure of yeast (Fig. S3B). Therefore, the higher ACC deaminase activity of SUTN9-2 (ACCDadap) was attributed to changes in the enrichment of the protein structure. Changes in enrichment also affected the electron density of the binding site of the ACC deaminase enzyme. Therefore, we suggested that the modification of the protein residue at Ser^140^ in the original strain to Asn^140^ in the improved strain affected the protein residue at Gly^161^ around the active site, resulting in a change in the inner diameter size of the cavity of the enzyme. Therefore, the change in the cavity of the enzyme resulted in an additional binding site in the SUTN9-2 (ACCDadap) strain (Table S2). Predicted binding site data showed conservations in amino acid residues contained in *Bradyrhizobium* sp. and *Pseudomonas* sp., and amino acid residues were also important for enzymatic activity. Based on the alignments of the three-dimensional structures, a small domain of ACC deaminase-containing bacteria (amino acid residues 58 to 169) may be the region responsible for the diversity or establishment of enzymatic activity by ACC deaminase-containing microorganisms (Jin *et al.*, 2016). Yao *et al.* (2000) proposed that amino acid residues 55 to 91, located on the small domain (helix 6) in the extra loop as a binding site of the ACC deaminase enzyme, were fixed after growth in medium broth supplemented with ACC for several selection rounds. However, this hypothesis needs to be clarified by site-directed mutation experiments in further studies. Since SUTN9-2 was isolated from *Aeschynomene americana* (Noisangiam *et al.*, 2012; Piromyou *et al.*, 2015), and it was confirmed as an endophyte in rice tissues by Piromyou *et al.* (2017), to develop SUTN9-2 strains as an inoculum using in the crop rotation between mung bean and rice, the bacterial strains will be evaluated in the aspect of endophytes in rice plant.

## Conclusions

ACC deaminase activity is an attribute of PGPR, which is dominant in the alleviation of stress ethylene under unfavorable conditions, such as water deficits. The improvement of ACC deaminase in SUTN9-2 using the ALE approach and genetic engineering increased ACC deaminase activity by SUTN9-2 (ACCDadap) and SUTN9-2:pMG103::*acdRS*, which may have enhanced the nodulation process. The high nodule numbers of mung bean plants inoculated with strains exhibiting increased ACC deaminase activity resulted in higher nodule dry weights and greater nitrogen-fixing efficiency at the seedling stages. Moreover, the negative effects of water deficits against nitrogen fixation may be attenuated by increasing the expression levels of the *acdS* and *acdR* genes in SUTN9-2:pMG103::*acdRS*. Moreover, the growth of and nitrogen fixation by mung bean plants inoculated with bradyrhizobia exhibiting increased ACC deaminase activity may be promoted under water deficit conditions. The present results suggest that the increased ACC deaminase activity of SUTN9-2:pMG103::*acdRS* affected ethylene synthesis by reducing ethylene concentrations. The present study demonstrated that high ACC deaminase activity may enhance symbiotic interactions, drought tolerance, and the reactivation of growth. Adaptive evolution using the ALE approach is a candidate strategy for further applications in the field.

## Citation

Sarapat, S., Songwattana, P., Longtonglang, A., Umnajkitikorn, K., Girdthai, T., Tittabutr, P., et al. (2020) Effects of Increased 1-Aminocyclopropane-1-Carboxylate (ACC) Deaminase Activity in *Bradyrhizobium* sp. SUTN9-2 on Mung Bean Symbiosis under Water Deficit Conditions. *Microbes Environ ***35**: ME20024.

https://doi.org/10.1264/jsme2.ME20024

## Supplementary Material

Supplementary Material

## Figures and Tables

**Table 1.** ACC deaminase activity of the *Bradyrhizobium* sp. SUTN9-2 strain and its developed strains, *Bradyrhizobium* sp. SUTN9-2 (wild-type strain), *Bradyrhizobium* sp. SUTN9-2 (ACCDadap) (adaptive strain), and *Bradyrhizobium* sp. SUTN9-2:pMG103::*acdRS* (increased copy number of *acdRS*).

Bacterial strains	ACC deaminase activity (μmol of α-ketobutyrate h^–1^ mg^–1^ protein)
SUTN9-2	3.88±0.16b
SUTN9-2 (ACCDadap)	5.58±0.08b
SUTN9-2:pMG103::*acdRS*	34.72±1.09a

Significance at *P*≤0.05. Data are shown as the mean±standard error (*n*=3).
