# Supplementary material for: Effects of Increased 1-Aminocyclopropane-1-Carboxylate (ACC) Deaminase Activity in Bradyrhizobium sp. SUTN9-2 on Mung Bean Symbiosis under Water Deficit Conditions
Source: Microbes Environ. 2020 Jun 16;35(3):ME20024. doi: 10.1264/jsme2.ME20024 (PMC7511786; doi:10.1264/jsme2.ME20024)
Supplement: Supplementary file 1 — Supplementary Material [file 35_20024_s1.pdf]

Supplementary information

Table S1 The number of bacterial cells in YEM containing 3.0 mM ACC for 0 day and 15 days

Bacterial strains	Number of bacterial cells (log10 CFU ml^{-1})		
	0 day	15 days	-fold
SUTN9-2:pMG103::*acdRS*	6.26±0.0364	9.11±0.0052a	1.21
SUTN9-2 (ACCDadap)	6.22±0.0310	8.14±0.0021b	1.08
SUTN9-2	6.17±0.0094	7.51±0.0195c	-

Data are average of three replicates ± standard error (S.E.); differing letters indicate significant differences at $P \leq 0.05$.

Table S2 The predicted binding site of ACC deaminase containing SUTN9-2 (ACCDadap) strain compared with its wild type strain (SUTN9-2) and two other strains, *Pseudomonas* including *P. putida* UW4 and *Pseudomonas* sp. ACP. The data were obtained from Phyre[2] software and analyzed by 3DLigandSite-Ligand binding site prediction server.

***Bradyrhizobium* sp. SUTN9-2**		***Bradyrhizobium* sp. SUTN9-2 SUTN9-2 (ACCDadap)**		***Pseudomonas putida* UW4 and *Pseudomonas* sp. ACP**	
Residue	Amino acid	Residue	Amino acid	Residue	Amino acid
50	ASN	50	ASN	50	ASN
51	LYS	51	LYS	51	LYS
54	LYS	54	LYS	54	LYS
79	ASN	79	ASN	79	ASN
-	-	*161	GLY	-	-
163	SER	163	SER	163	SER
196	CYS	196	CYS	196	CYS
197	THR	197	THR	197	THR
198	VAL	198	VAL	198	VAL
199	THR	199	THR	199	THR
200	GLY	200	GLY	200	GLY
201	SER	201	SER	201	SER
202	THR	202	THR	202	THR
294	TYR	294	TYR	294	TYR
295	GLU	295	GLU	295	GLU
322	LEU	322	LEU	322	LEU
323	GLY	323	GLY	323	GLY
324	GLY	324	GLY	324	GLY

***** is a predicted binding site of AcdS protein which was only plus in the adaptive bacterial strain, SUTN9-2 (ACCDadap), and it was the effects of the nucleic acid sequence and amino acid residue changes.

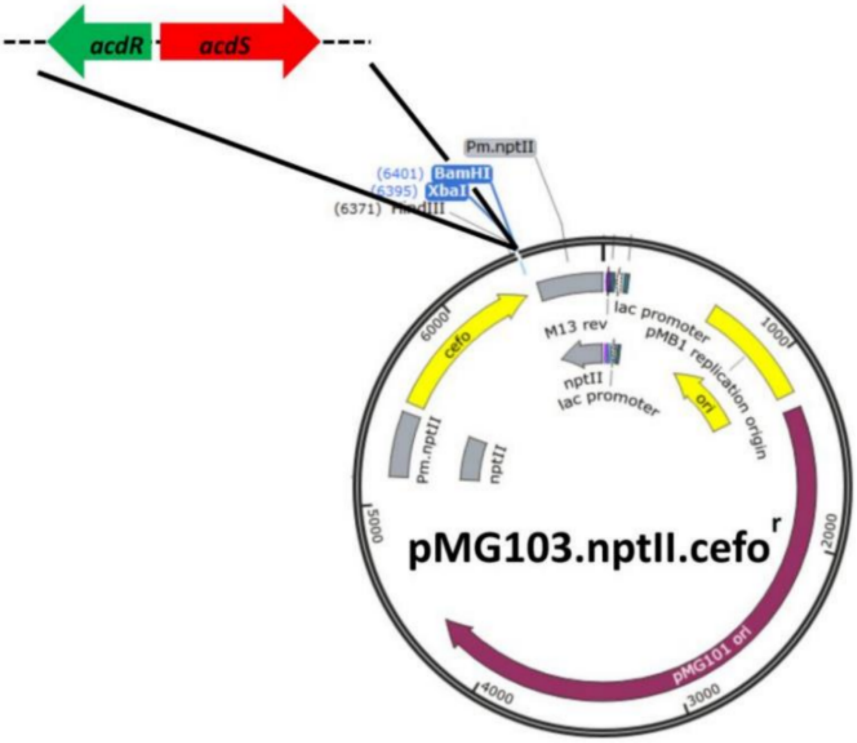

Fig. S1 Physical map of vector containing *acdR* and *acdS* fragment was amplified from *Bradyrhizobium* sp. SUTN9-2 genome, the fragment gene was inserted between the cefotaxime resistant gene (cefo^{r}) and neomycin phosphotransferase II gene (nptII) of pMG103 vector.

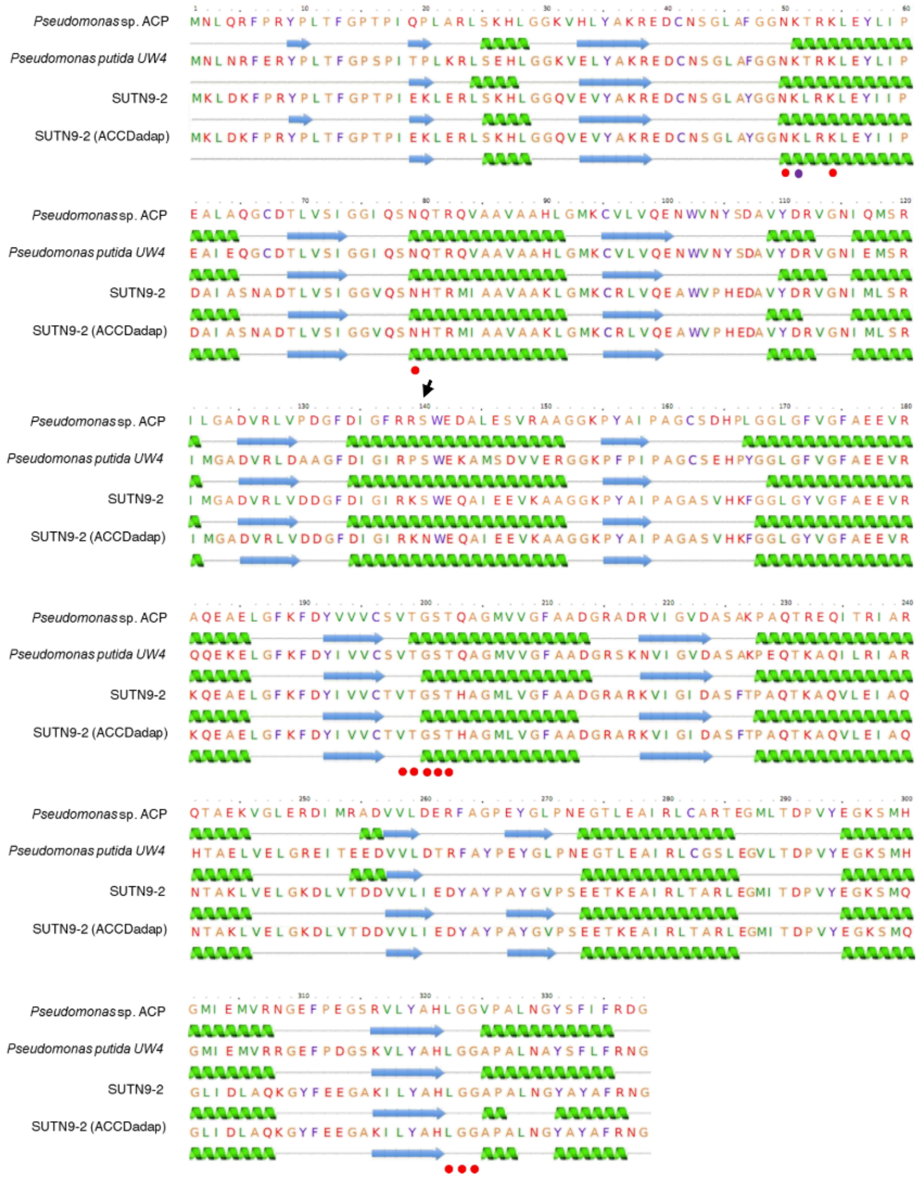

Fig. S2 The secondary protein structures of ACC deaminase containing *Bradyrhizobium* sp. SUTN9-2 (ACCDadap) compared with SUTN9-2 WT and two other strains of *Pseudomonas*, including *Pseudomonas putida* UW4 and *Pseudomonas* sp. ACP. The red circles are the predicted binding site of amino acid residues of ACC deaminase that were predicted by Phyre^2 software and analyzed by 3DLigandSite-Ligand binding site prediction server. The arrow is a position of amino acid residue that changed from serine (Ser/S) in SUTN9-2 wild type to be an asparagine (Asn/N) in SUTN9-2 (ACCDadap) at the 140 residue region.

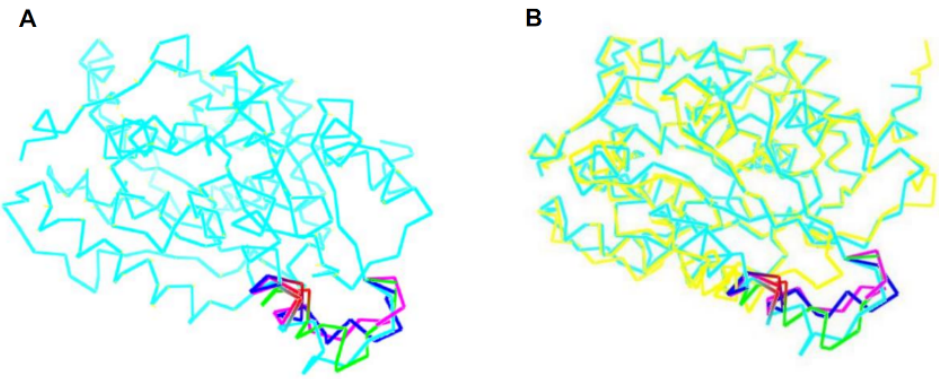

Fig. S3 The three-dimensional structures of ACC deaminase enzyme display in different colors. (A) The superposition of ACC deaminase structure of SUTN9-2 WT (aqua color) and SUTN9-2 (ACCDadap) (green color) compared with the other bacteria, *P. putida* UW4 (blue color) and *Pseudomonas* sp. ACP (pink color) in ribbon model. (B) The superposition of ACC deaminase structure of SUTN9-2 WT (aqua color) and SUTN9-2 (ACCDadap) (green color), *P. putida* UW4 (blue color) and *Pseudomonas* sp. ACP (pink color) compared with yeast, *Hansenula saturnus* (yellow color).
